# Initial experience of a novel surgical assist robot “Saroa” featuring tactile feedback and a roll-clutch system in radical prostatectomy

**DOI:** 10.1038/s41598-024-82531-3

**Published:** 2024-12-30

**Authors:** Kosuke Iwatani, Fumihiko Urabe, Shun Saito, Shota Kawano, Tomoya Yamasaki, Shoji Kimura, Hideo Otsuki, Kei Fujio, Takahiro Kimura, Jun Miki

**Affiliations:** 1 Department of Urology, The Jikei University School of Medicine, Kashiwa Hospital, Kashiwashita 163-1, Kashiwa, Chiba 277-8567 Japan; 2 Department of Urology, The Jikei University School of Medicine, Tokyo, Japan; 3 Department of Urology, The Jikei University School of Medicine, Daisan Hospital, Chiba, Japan; 4 Department of Urology, Abiko Toho Hospital, Chiba, Japan

**Keywords:** Robot-assisted radical prostatectomy, Tactile feedback, Saroa, Roll-clutch, Cancer therapy, Prostate cancer, Mechanical engineering, Surgical oncology

## Abstract

To evaluate the safety and efficacy of the Saroa Surgical Robot System in robot-assisted laparoscopic radical prostatectomy (RARP). We enrolled 60 patients who underwent RARP using either the Saroa (n = 9) or da Vinci Xi (n = 51) systems at Jikei University Kashiwa Hospital from January 2022 to March 2024. We compared preoperative characteristics, perioperative outcomes, complications, and postoperative urinary continence at three months between the two groups. No significant differences were found in preoperative characteristics. The Saroa group had a longer median operative time compared to the da Vinci group. Postoperative urinary continence rates were slightly lower in the Saroa group (77.8 % vs. 84.6%), though not statistically significant. When the tactile feedback function was activated, the organs were grasped with less force compared to when it was off. This study is the first to assess the Saroa system’s effectiveness and safety in RARP. While the system shows promise, especially with tactile feedback that aids in delicate tissue handling, further investigation is needed to evaluate long-term oncological and functional outcomes.

## Introduction

Robotic surgical systems have revolutionized laparoscopic surgery, offering enhanced articulation, dexterity, and precision for complex procedures, such as radical prostatectomy^1^. These systems enable surgeons to operate more intuitively, with reduced endoscope tremors and enhanced accuracy^2^. However, the risk of injury and hemorrhage associated with excessive force during tissue and organ retraction remains a concern^3^. Therefore, a novel robotic system known as Saroa (Riverfield Inc., Tokyo, Japan) has been developed. Saroa has a compact design with three pneumatically powered arms that directly transmit the tactile sensation of forceps pressure to the surgeon; moreover, it incorporates a unique roll-clutch system for effortless wrist control. Although it has demonstrated utility in colorectal cancer resection^4^, our study is the first to evaluate its effectiveness and safety in robot-assisted laparoscopic radical prostatectomy (RARP). We compared preoperative characteristics, perioperative and pathological outcomes, complications, and postoperative urinary continence between the two groups, who underwent RARP using Saroa or da Vinci Xi (Intuitive Surgical, Sunnyvale, CA, USA).

## Methods

We enrolled 60 patients who underwent RARP using Saroa (n = 9) or da Vinci Xi (n = 51) at Jikei University Kashiwa Hospital between January 2022 and March 2024. Procedures were performed by KI and JM (experience with >50 da Vinci Xi cases). The da Vinci Xi system was used between January 2022 and March 2024, and Saroa was used between August 2023 and March 2024. We excluded 25 cases that involved lymphadenectomy. Preoperative characteristics, perioperative and pathological outcomes, complications, and postoperative urinary continence at 3 months after were compared between the two groups. A single pathologist assessed all prostate specimens. Postoperative continence was defined as requiring 0–1 security pads per day. The Institutional Review Board of the Jikei University School of Medicine [No. 35–398(12035)] approved the study protocol. RARP using Saroa was performed after obtaining approval from the Highly Difficult Technology Committee and the Medical Safety Committee at our hospital, and informed consent was obtained from all participants. Patients in the Saroa group provided consent after being fully informed that RARP with Saroa was performed exclusively at this hospital and that the device’s safety had been thoroughly reviewed and approved beforehand.

### Saroa platform features

**Fig. 1:**
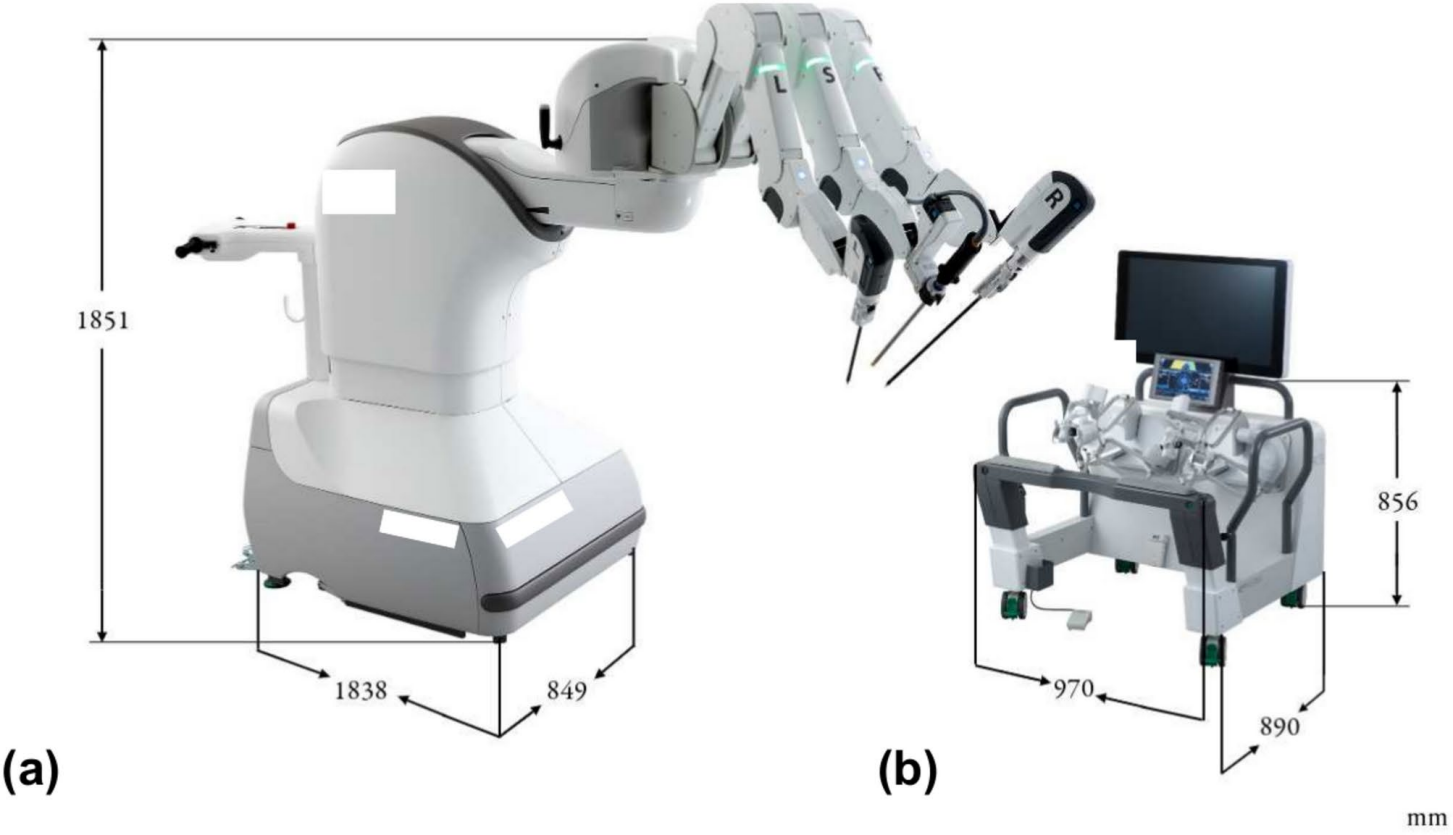
Saroa Surgical System. (**a**) Patient Cart. (**b**) Surgeon console with 3D–4K monitor.

**Fig. 2:**
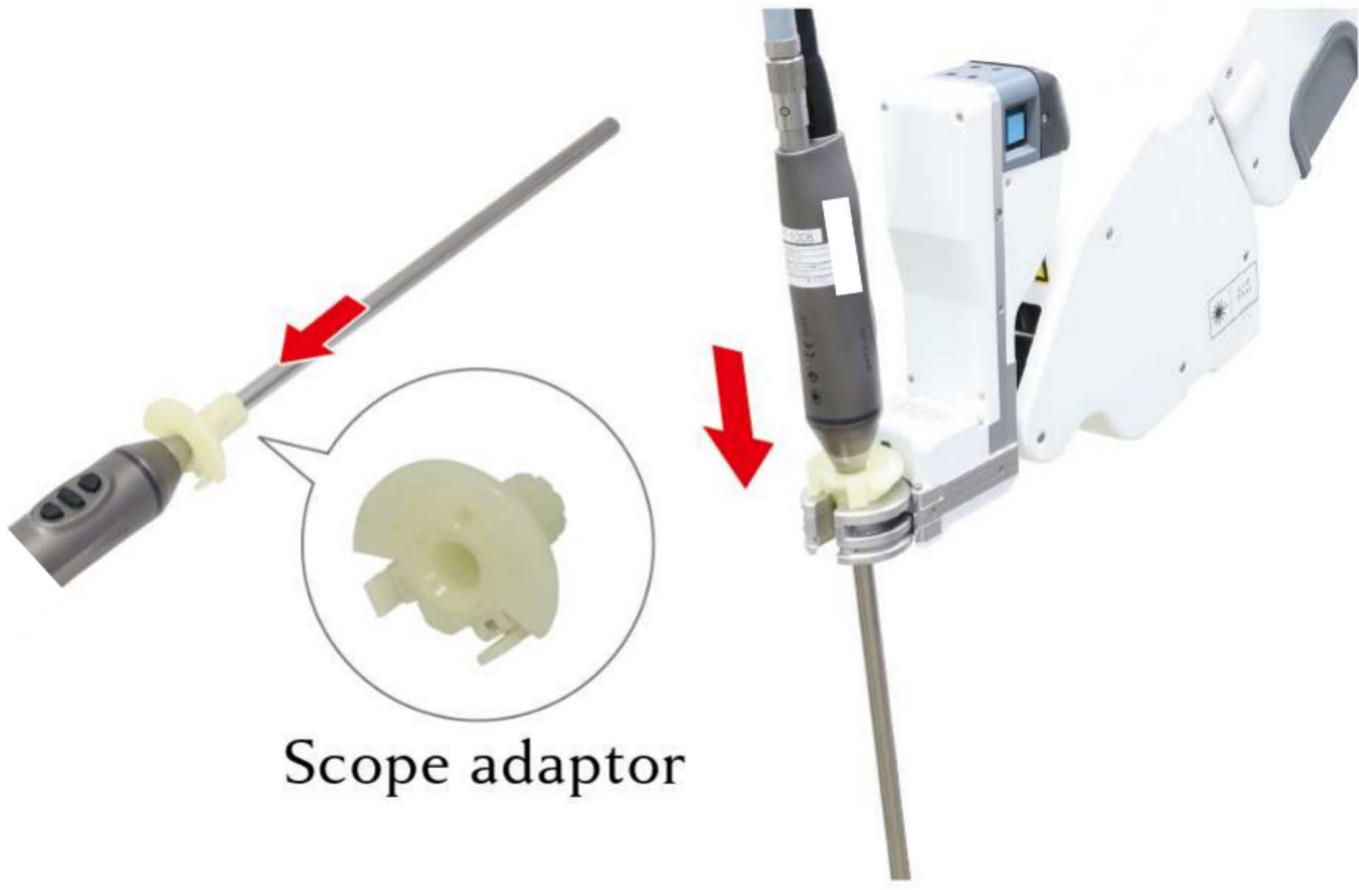
Endoscope adaptor. Attach an adapter to the endoscope shaft and connect it to the camera arm.

**Fig. 3:**
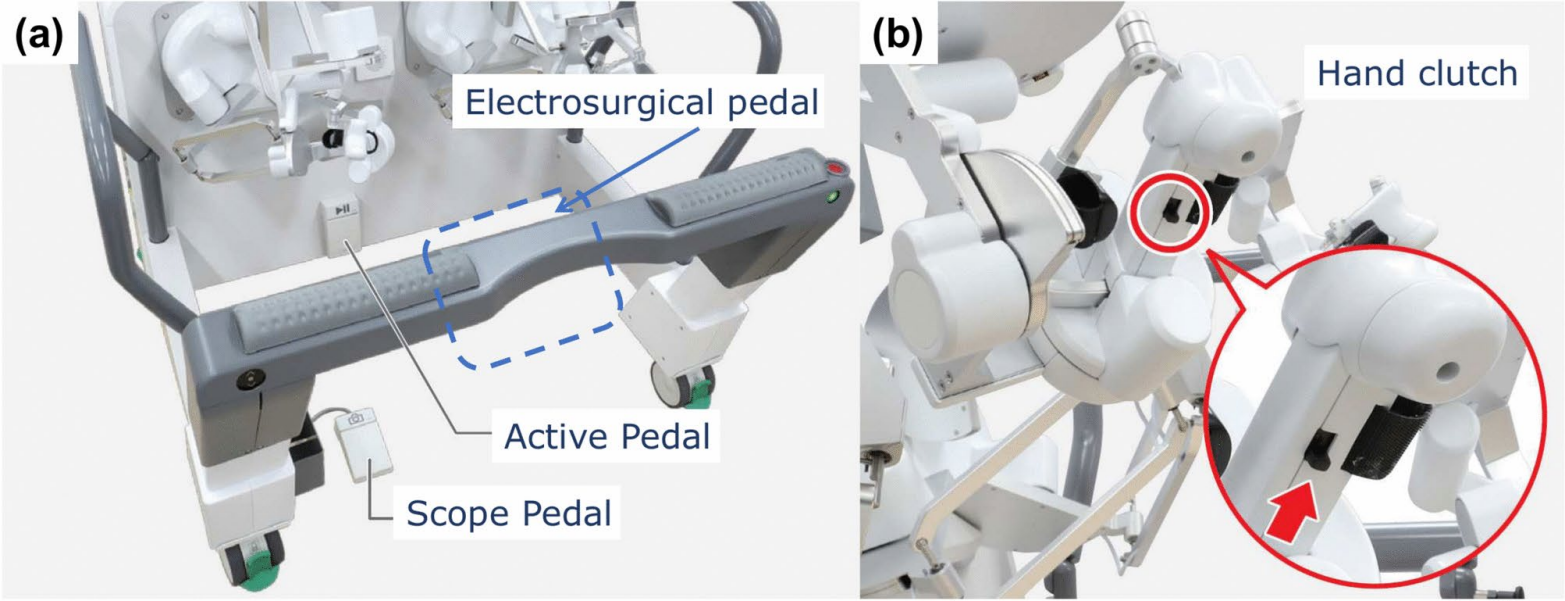
Surgeon Console. (**a**) Foot Pedals: The scope pedal and hand controller are used to maneuver the endoscope, while the active pedal is pressed to operate Saroa. Electrosurgical pedals are installed for each device. (**b**) Hand Controller: The operating fingers are the thumb and index finger, while the middle finger is used for the hand clutch switch.

Saroa is a three-armed robot that includes a patient cart and an open console for the surgeon. Compared to other models, Saroa is compact and lightweight, with the patient cart weighing 492 kg and the surgeon console 130 kg (Fig. 1). The robot’s left and right arms hold surgical instruments, while the center arm holds an endoscope. Different types of endoscopes can be attached using the adapter (Fig. 2). Like other master-slave robots, the surgeon operates from the console, controlling the camera with a footswitch and hand controls (Fig. 3). For oblique endoscopes, switching between up and down views is done manually by simply rotating the endoscope, which is quick and easy.

**Fig. 4:**
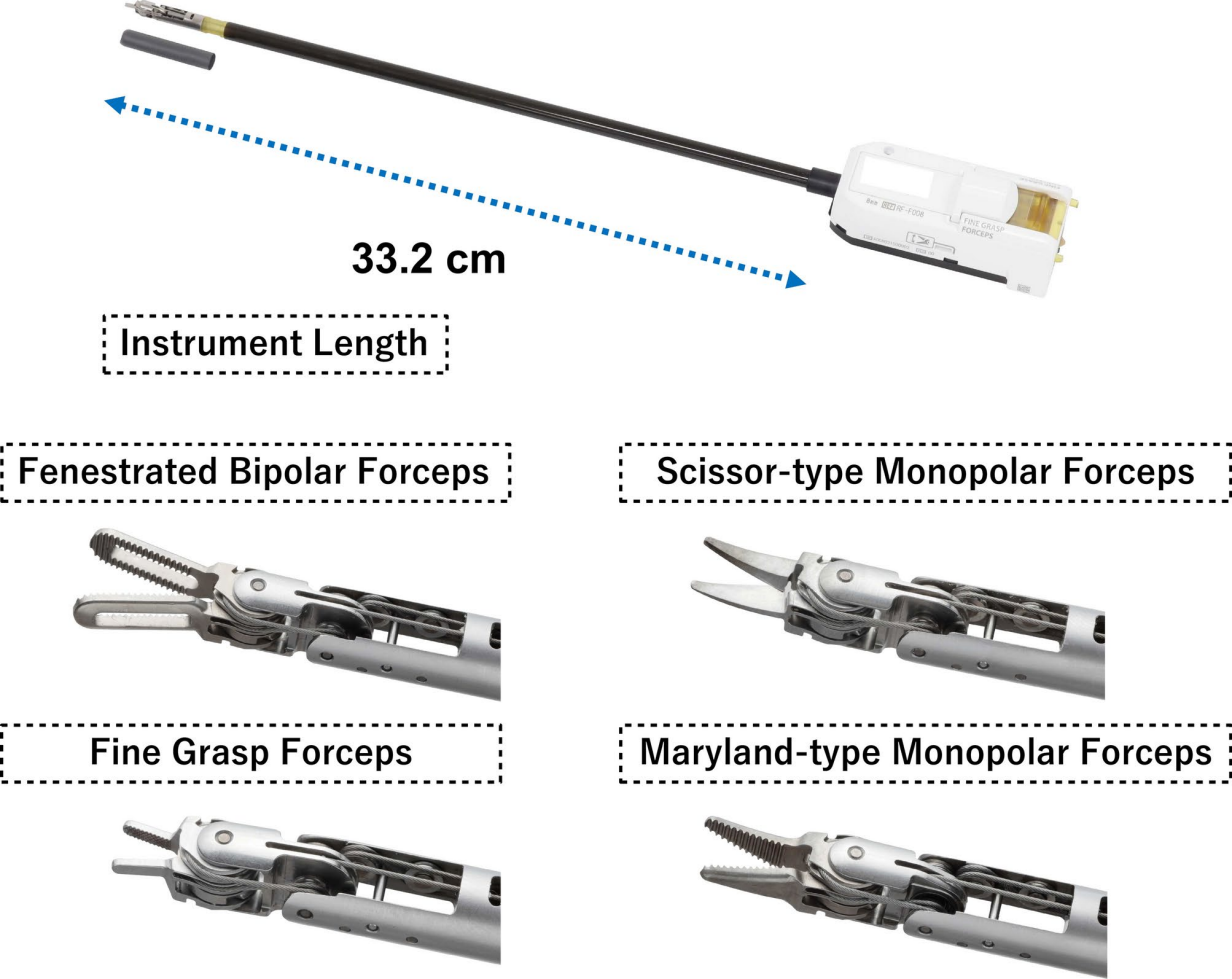
Shape and type of forceps. The shaft of Saroa’s forceps is slightly thicker and 33.2 cm long. 4 different types of forceps are used in the surgery.

The left hand typically uses fenestrated bipolar forceps, while the right hand uses either scissor-type or Maryland-type monopolar forceps. Fine grasp forceps are used for needle driving (Fig. 4). Electrosurgical devices can be used as long as they are compatible. We used ERBE VIO 300 D (ERBE USA, Marietta, GA) and Valleylab™ FT10 (Medtronic, Minneapolis, MN, USA) electrosurgical generators. Saroa’s forceps are shorter compared to da Vinci (Saroa 33.2 cm, da Vinci 56.5 cm), and the forceps are slightly thicker to support pneumatic drive system, requiring 11-12 mm trocars, but no dedicated trocar system is necessary as it’s docking-free. By tracking the coordinates of the abdominal wall during forceps insertion, the robot arm can pivot around the abdominal wall as its fulcrum.

**Fig. 5:**
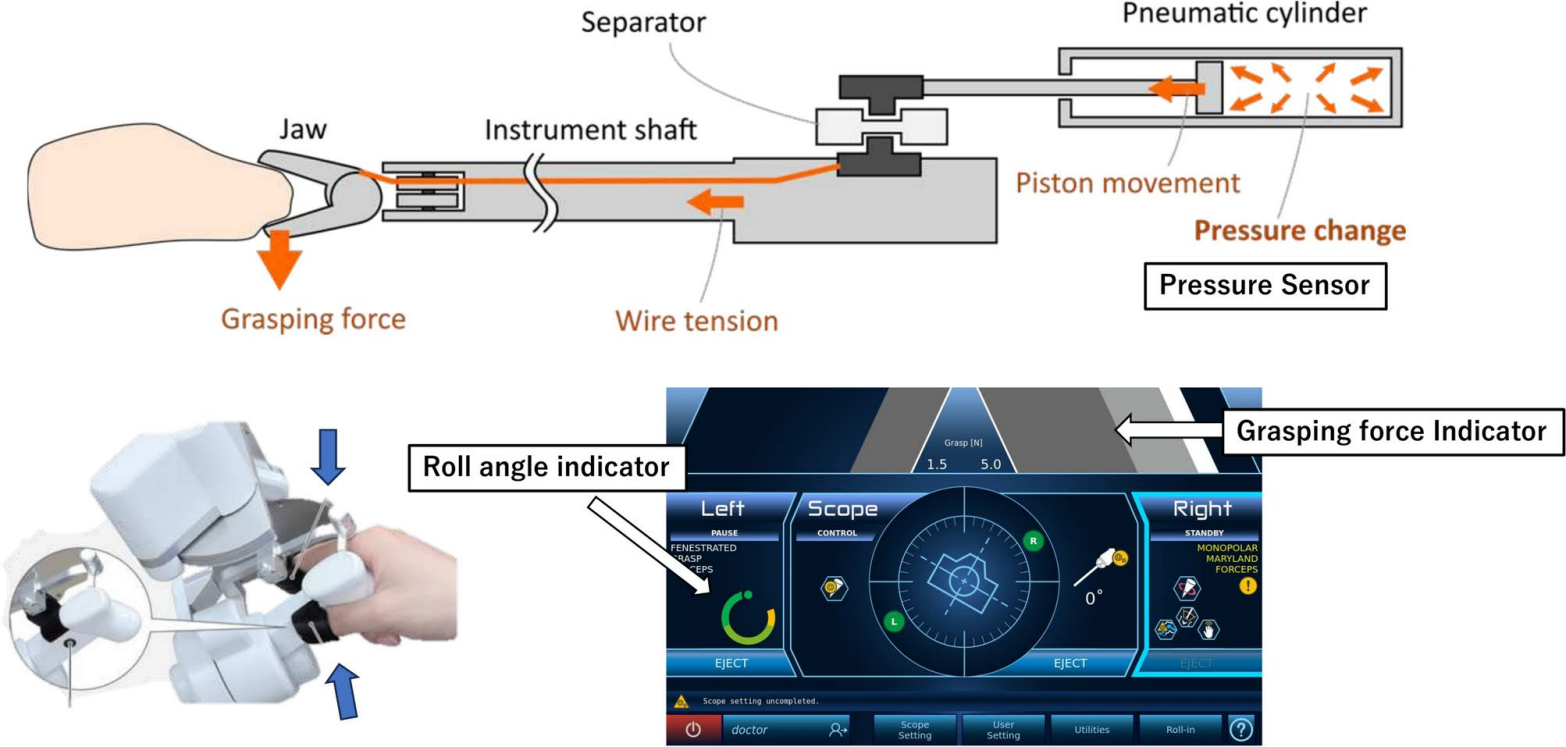
Pneumatic drive and tactile feedback. When an object is grasped, the force is perceived as air pressure through the cylinder, providing tactile feedback. The grasping force is displayed in real time on the indicator of the sub-monitor, and the function can be turned on or off using the sub-monitor. The status of roll-clutch rotation is also displayed.

**Fig. 6:**
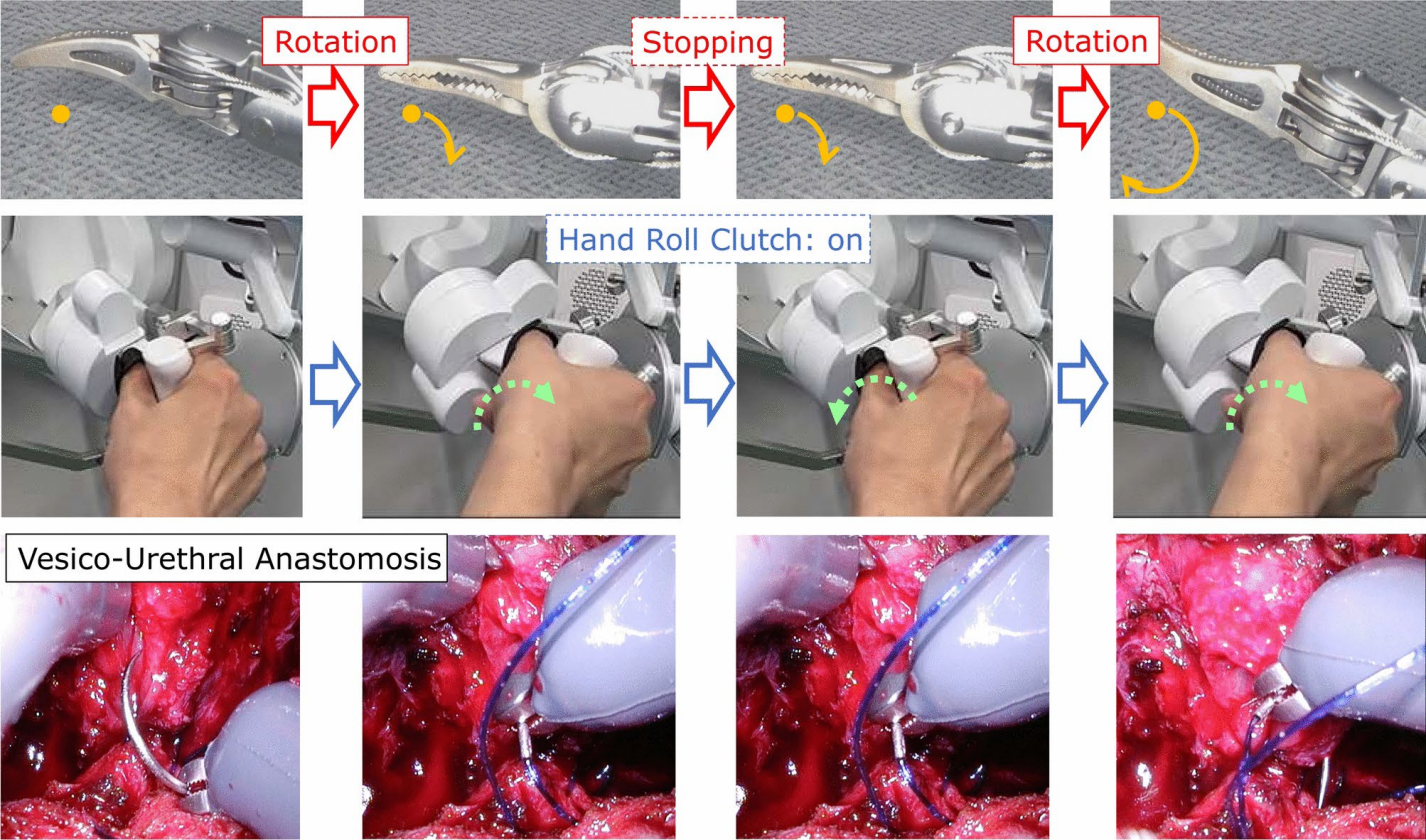
Roll-clutch system. Surgeon wrist rotation with clutch activation allows 290 degrees of rotation in each direction (580 degrees total), exceeding the range of motion offered by other robotic systems.

Additionally, Saroa’s distinctive features include tactile feedback and a roll-clutch system. Saroa uses a pneumatically driven system that allows the surgeon to feel the force feedback by the robot when grasping objects (Fig. 5). This feature can be turned on or off as needed, and all grasping forces during surgery are recorded in real-time. The roll-clutch system enables the surgeon to reset wrist rotation through clutch operation, allowing the wrist to rotate 290 degrees in both clockwise and counterclockwise directions (Fig. 6). This ensures that the surgeon can always operate with a comfortable wrist angle.

### Surgical procedure

**Fig. 7:**
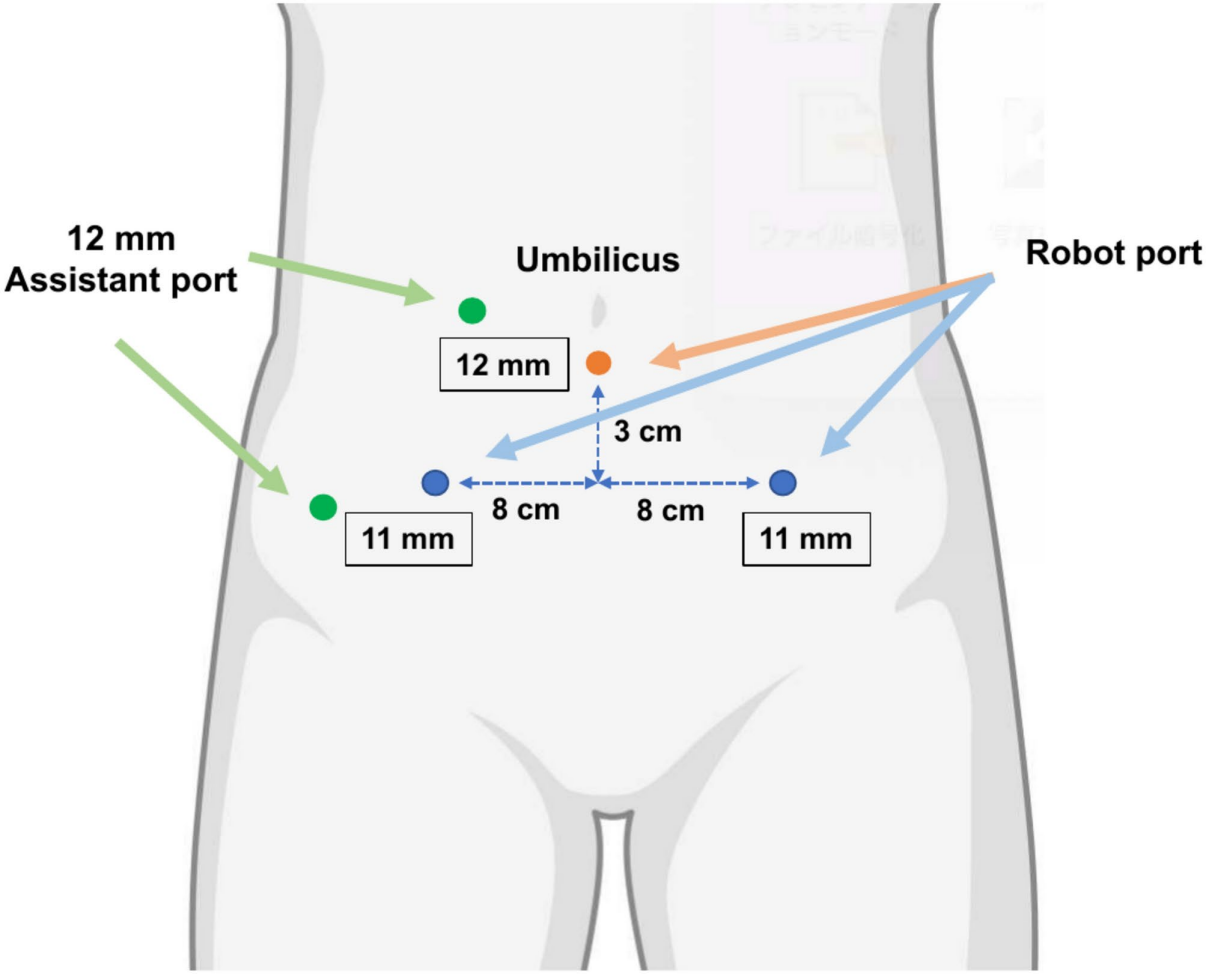
Port arrangement. The camera port is positioned below the umbilicus, with the left and right ports positioned 8 cm apart.

**Fig. 8:**
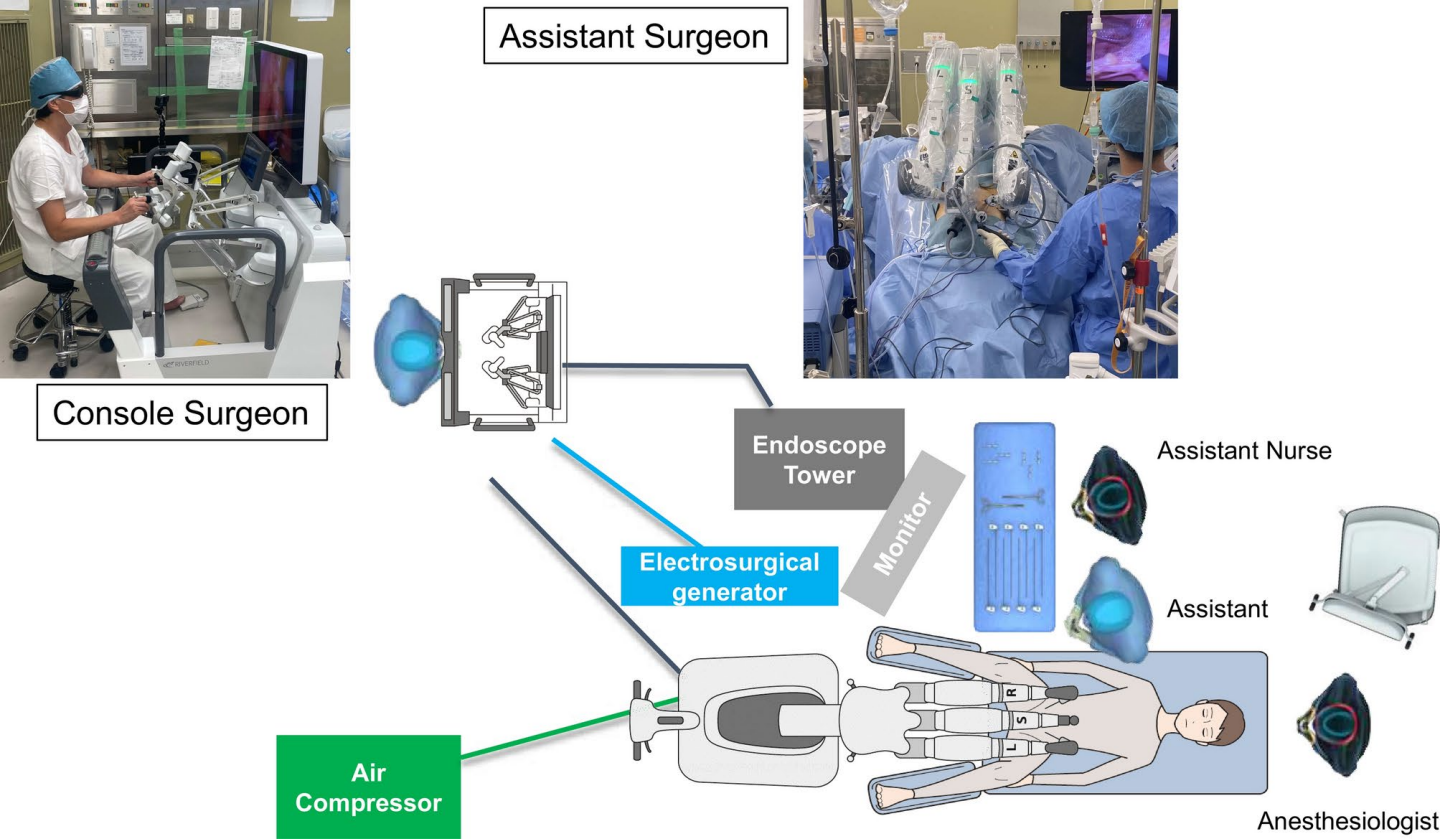
Operation setup. The assistant positioned on the right side of the patient. The Saroa Surgical System requires an external electrosurgical generator (located between the console and Saroa).

RARP was performed in a 25° head-down supine position (da Vinci system) or the lithotomy position (Saroa Surgical System). Saroa has one less arm than the da Vinci, so it takes up less space for lateral ports. The camera port was placed below the umbilicus to give the assistant more working space (Fig. 7). The assistant stood on the patient’s right side and maneuvered the Saroa robot into position from the patient’s caudal side (Fig. 8). We used a trans-peritoneal approach with bladder neck preservation. Nerve-sparing decisions followed thorough patient consultation. Vesicourethral anastomosis was performed using continuous 3–0 Stratafix (Covidien, Mansfield, MA, USA) sutures.

### Statistical analysis

Patient characteristics and perioperative outcomes, perioperative complications and postoperative urinary continence were compared using the Mann-Whitney U and chi-square tests. Statistical analyses were performed using R software (version 4.20, R Foundation for Statistical Computing, Vienna, Austria). P-values < 0.05 were considered statistically significant.

## Results

### Treatment outcomes

Table 1 presents the baseline characteristics of the study participants. No significant differences were observed between the groups in preoperative demographics, such as patient age, body mass index, baseline serum levels of PSA, and prostate volume. Table 2 summarizes the perioperative outcomes. Nerve-sparing procedures were performed in 13 patients (50%) in the da Vinci group. The Saroa group demonstrated significantly longer operative and console times (median operative time: 273 [247–290] min vs. 218 [186–230] min, P < 0.001; median console time: 195 [190–226] min vs. 136 [120–162] min, P < 0.001). No significant differences were found in other surgical outcomes, including estimated blood loss, intraoperative blood transfusion, intraoperative complications, or major postoperative complications (defined by the Clavien-Dindo classification). Surgical positive margin rates and other pathological outcomes were similar between the groups. There was no significant difference in urinary continence at three months postoperatively, although the da Vinci group had slightly better outcomes (77.8% vs. 84.6%).

**Table 1:** Patinent demongraphics.

	Saroa group	da Vinci group	p-value
No of patients	9	26	
Age, median (IQR)	71 (63–77)	72 (69–75)	0.698
BMI, median (IQR)	24.9 (22.0–25.5)	23.9 (22.0–26.5)	0.91
Baseline PSA level (ng/dl), median (IQR)	7.15 (6.40–11.56)	9.61 (6.15–13.45)	0.651
Gleason grade group, n (%)			
1	0 (0.0)	2 (7.7)	0.856
2	2 (22.2)	9 (34.6)	
3	4 (44.4)	6 (23.1)	
4	1 (11.1)	3 (11.5)	
5	2 (22.2)	6 (23.1)	
D’Amico risk classification, n (%)			
Low	0 (0.0)	0 (0.0)	1
Intermediate	5 (55.6)	14 ( 53.8)	
High	4 (44.4)	12 ( 46.2)	
Prostate volume (cm3), median (IQR)	26.3 (23.0-40.0)	30.0 (27.0–48.3)	0.173

IQR interquartile range.

**Table 2:** Perioperative outcomes.

	Saroa group	da Vinci group	p-value
No of patients	9	26	
Nerve sparing, n (%)	0 (0.0)	13 (50.0)	0.013
Lymph node dissection, n (%)	0 (0.0)	0 (0.0)	NA
Blood trans fusion, n (%)	0 (0.0)	0 (0.0)	NA
Estimated blood loss (ml), median (IQR)	50 (50–150)	50 (50–150)	0.797
Operation time (min), median (IQR)			
Console time	195 (190–226)	136 (120–162)	<0.001
Total operation time	273 (247–290)	218 (186–230)	<0.001
Intraoperative complication, n (%)	0 (0.0)	0 (0.0)	NA
Postoperative complication, n (%)			
None	8 (88.9)	22 (84.6)	1
Grade I	0 (0.0)	1 (3.8)	
Grade II	1 (11.1)	2 (7.7)	
Grade IIIa	0 (0.0)	1 (3.8)	
pTstage, n (%)			
pT2a,b	5 (55.6)	17 (65.4)	0.698
pT3a,b	4 (44.4)	9 (34.6)	
EPE, n (%)			
Negative	6 (66.7)	16 (61.5)	1
Positive	3 (33.3)	10 (38.5)	
RM (%)			
Negative	6 (66.7)	18 (69.2)	1
Positive	3 (33.3)	8 (30.8)	
Continence rate at after 3 month, n (%)	7 (77.8)	22 (84.6)	1

Complications are defined by Clavien-Dindo classification.

*EPE* extra prostatic extension, *RM* resection margin, *IQR* interquartile range.

### Surgical feasibility

**Fig. 9:**
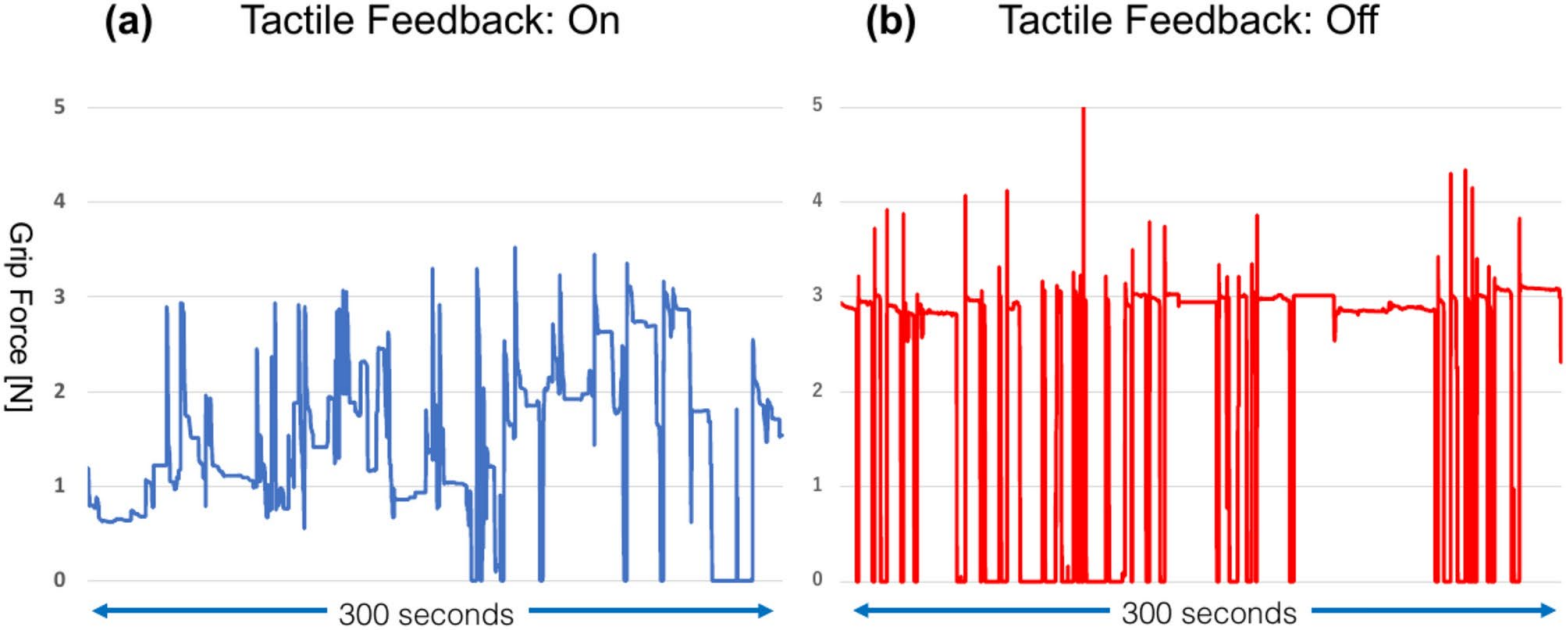
Tactile feedback. (**a**) Tactile feedback is enabled. (**b**) Tactile feedback is disabled. Tactile feedback allows for object manipulation with reduced gripping force.

**Fig. 10:**
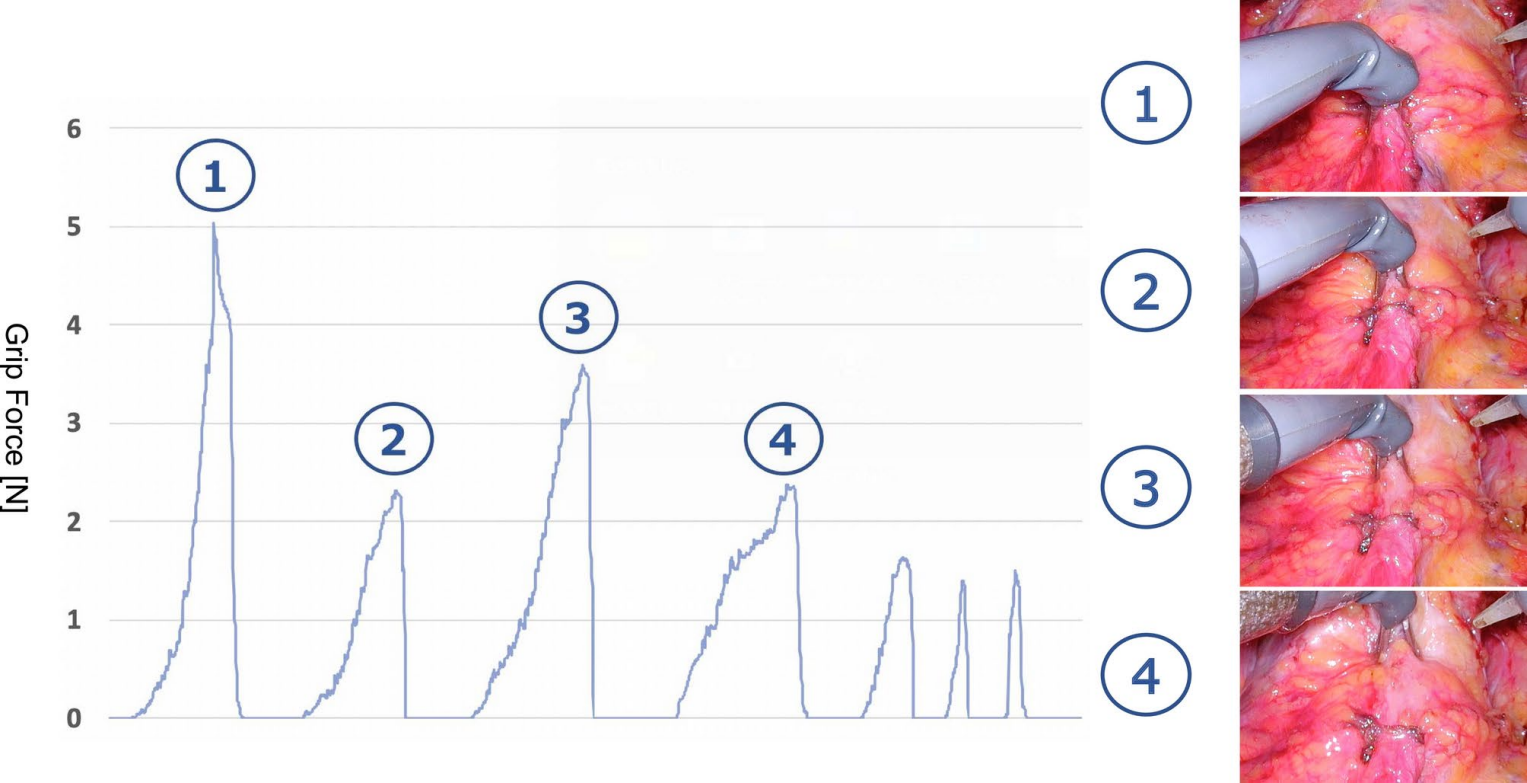
Tactile feedback in bladder-prostate area. The graph demonstrates the grasping force required to maintain consistent forceps opening while moving from the bladder to the prostate (1→4).

The Saroa system integrates novel features, including tactile feedback and a roll-clutch system. We measured the grasping force exerted by the robot on organs with and without tactile feedback. Tactile feedback significantly reduced the overall force, indicating that surgeons may apply excessive force without this sensory input (Fig. 9). In addition, force measurements with equal forceps closure revealed significant differences between the bladder and prostate (Fig. 10), emphasizing the inherent difficulty of visually differentiating tissue boundaries. The roll-clutch system proved essential for suturing, enabling deep stitches without re-holding the needle. The clutching mechanism minimized force on the puncture site during needle repositioning.

## Discussion

Robotics-assisted surgery offers several advantages, including tremor reduction, enhanced precision, shorter learning curves, and improved surgical and oncological outcomes^5–7^. Robotic surgery has significantly impacted radical prostatectomy, combining the advantages of open and laparoscopic techniques while enabling precise manipulation at the pelvic floor. We evaluated the effectiveness and safety of Saroa, a novel surgical assistant with tactile feedback, in RARP.

Recent advancements in surgical robots prioritize replicating the dexterity of surgeon hand movements during minimally invasive procedures^8,9^. However, tissue damage caused by excessive force during organ manipulation remains a critical concern. Wottawa et al. investigated the impact of tactile feedback on novice and expert robotic surgeons, focusing on its effect on grasping forces and organ damage. Their findings revealed that effective tactile feedback led to a significant reduction in grasp force and tissue injury for both groups, along with a notable correlation between grasp strength and tissue damage. Additionally, many participants reported that tactile feedback enhanced their performance and awareness during delicate grasping^10^. This is particularly beneficial for surgeons with limited laparoscopic experience, allowing gentler tissue handling^11^. The Saroa system also addresses this challenge by incorporating tactile feedback, allowing surgeons to perceive resistance while grasping. Although a firm grasp may be necessary for certain instruments, such as needles, this can lead to finger fatigue. Notably, the grasping force required differs significantly between prostate and bladder tissues (Fig. 4); this emphasizes a key advantage over the da Vinci system, which primarily relies on visual cues for bladder neck dissection. Using Saroa, the bladder neck can be recognized using visual and tactile feedback. This dual-sensory approach could broaden its utility in various surgeries. Furthermore, the ability to determine the exact dissection plane through tactile grasping may help surgeons perform higher-quality surgeries.

Saroa incorporates several innovative features designed to enhance precision and workflow in minimally invasive surgery. One key feature is a roll-clutch system that facilitates highly precise needle movements, particularly during deep, delicate insertions. Unlike conventional robotic platforms with limited arm rotation, Saroa permits continuous needle driving, minimizing tissue damage. This enhanced control improves needle handling and allows surgeons to perform a wider range of surgical maneuvers. Furthermore, its open console design promotes better communication between the operating surgeon and the surgical team, enabling multiple surgeons to visualize the procedure simultaneously in 3D.

In our study, the operation took longer using Saroa. This might be attributable to the unfamiliarity of surgeons with the system vs. da Vinci Xi and the absence of a fourth robotic arm. The fourth arm plays a crucial role in procedures such as RARP, which provides necessary tension on pelvic floor organs. This function must be performed by an assistant surgeon when using Saroa, increasing operation time due to reduced coordination in early robotic surgeries; however, surgeon–assistant coordination is improving with practice. Moreover, plans are underway to introduce two more arms. However, the current three-arm design offers advantages in weight and size, simplifying installation in operating rooms of varying dimensions. Furthermore, its compatibility with standard endoscopes, trocars, and equipment significantly reduces operating costs compared to conventional systems.

Postoperative incontinence was slightly higher in the Saroa group. The two patients who experienced urinary incontinence at three months after were cases 1 and 3, likely due to the surgeon’s unfamiliarity with the Saroa and the initial lack of smooth movement and insufficient force from the Saroa forceps, which did not adequately preserve the prostatic apex muscles. We have made several software and hardware updates based on feedback from each case. The current version is easier to handle and has shown better urinary continence results, particularly due to enhanced force and accuracy of the forceps.

Our study is the first to evaluate the effectiveness and safety of Saroa in RARP. Although surgeons experienced in robotic surgery performed the procedures safely, operative times were slightly extended. Implementing surgery with a new robot involves repeated trial and error in port placement and surgical strategy based on the robot’s characteristics. This report describes the world’s first urologic surgery performed using the Saroa system and highlights the process of reviewing each case and efforts made to improve surgical outcomes. Further investigation is warranted to assess long-term oncological and functional outcomes following RARP with Saroa.

## Data Availability

The datasets used and/or analysed during the current study available from the corresponding author on reasonable request.

## Abbreviation

RARP: Robot-assisted radical prostatectomy

## Declarations

### Approval of the research protocol by an institutional reviewer board

The Ethics Committee of the Institutional Review Board of the Jikei University School of Medicine [No. 35–398(12035)] approved the study protocol. The study was performed in accordance with the Declaration of Helsinki.
